# *In vivo* antitumour potential of camel’s milk against hepatocellular carcinoma in rats and its improvement of cisplatin renal side effects

**DOI:** 10.1080/13880209.2017.1309553

**Published:** 2017-04-04

**Authors:** Hala M. F. El Miniawy, Kawkab A. Ahmed, Sameeh A. Mansour, Marwa M. Salah Khattab

**Affiliations:** a Department of Pathology, Faculty of Veterinary Medicine, Cairo University, Giza, Egypt;; b Department of Pesticide Chemistry, National Research Center, Giza, Egypt

**Keywords:** Diethylnitrosamine, placental glutathione-S-transferase, superoxide dismutase, lipid peroxidation, histopathology

## Abstract

**Context:** Camel milk (CM) is recommended for liver disease patients in Egypt for a strong belief that it has a curative effect.

**Objective:** The effect of consumption of CM with or without chemotherapeutic drug cisplatin was evaluated on induced hepatocarcinogenesis in rats.

**Materials and methods:** Wistar male rats (56) were divided into eight groups (7 rats each). Group I was control. Hepatocarcinogenesis was initiated by a single dose of intraperitoneal injection of diethylnitrosamine (DENA) (200 mg/kg BW) and promoted by phenobarbitone (500 ppm) in drinking water in groups V, VI, VII and VIII. Treatment started from 28th till 38th week using CM (5 mL/day) and/or cisplatin (5 mg/kg/3 weeks) in groups II, III IV, VI, VII and VIII. Biochemical analysis, lipid peroxidation and superoxide dismutase (SOD) activity in liver tissue were performed. Histopathology of liver and kidney and immunohistochemistry of placental glutathione-*S*-transferase (P-GST) in liver were performed and analyzed using image analysis.

**Results:** Albumin concentration and SOD activity were 3.13 ± 0.23 and 311.45 ± 41.71 in group VII (DENA & cisplatin), whereas they were 4.3 ± 0.15 and 540.5 ± 29.94 in group VII (DENA, CM and cisplatin). The mean area of altered hepatocellular foci and P-GST altered foci decreased in group VI (DENA and CM) (1049.6 ± 174.78 and 829.1 ± 261) and group VIII (cisplatin and CM) (1615.12 ± 436 and 543.9 ± 127) compared to group V (DENA only) (4173.74 ± 510.7 and 3169.49 ± 538.61). Cisplatin caused chronic interstitial nephritis, which was slightly alleviated in group VIII (CM and cisplatin).

**Conclusions:** CM had an antioxidant effect and together with cisplatin managed to decrease hepatocarcinogenesis.

## Introduction

Primary liver cancer, also known as hepatocellular carcinoma (HCC), is a disease with an extremely poor prognosis and a 5-year survival rate below 9% (Sherman 2005). Many risk factors have been implicated in the occurrence of HCC including contamination of foods with mycotoxins, hepatitis viral infections, exposure to genotoxic and cytotoxic chemicals and high levels of alcohol consumption which in turn results in chronic liver injury, inflammation and oxidative stress (Klaunig & Kamedulis 2004; Gao et al. 2012).

Cisplatin is the prototype of the chemotherapy class of platinum drugs. They cause cell death by binding to DNA to form DNA adducts, preventing further replication (Arnesano & Natile 2009). Cisplatin-based combination chemotherapy regimens are currently used in the treatment of patients with a solid tumour (Langerak & Dreisbach 2001) never the less cisplatin was found to induce serious side effects including nephrotoxicity, neurotoxicity, ototoxicity, nausea and vomiting (Giaccone 2000).

Camel’s milk (CM) is an excellent source of well-balanced nutrients (Gorban & Izzeldin 2001) and also exhibits a wide range of biological activities; antimicrobial, antioxidative, antithrombotic, antihypertensive and immuno-modulatory effect (FitzGerald & Meisel 2000; Kohonen & Pihlanto 2003; Saltanat et al. 2009). These biological activities are mainly due to the presence of peptides and protein in milk (Kohonen & Pihlanto 2001). Hepatoprotection due to the administration of camel milk has been previously documented (Sharmanov et al. 1982; Darwish et al. 2012). In Egypt, camel milk obtained from free-ranging desert camels is being consumed by chronic liver disease patients based on the belief that it improves their liver function. A few studies showed that camel milk reduced the survival and proliferation of tumour cell lines through the activation of both the extrinsic and intrinsic apoptotic pathways (Korashy et al. 2012b). Concomitantly, our previous work showed a positive effect of camel milk on induced hepatocarcinogenesis (El Miniawy et al. 2014). Therefore, this study assesses the effect of consuming camel milk either alone or combined with cisplatin against hepatic cancer.

## Materials and methods

### Animals

Fifty-six male Wistar rats, weighing 100–120 g, were purchased from the animal house of the National Research Center (El Dokki, Giza, Egypt). This experimental work was approved by Ethics of Animal Use in Research Committee (EAURC), Faculty of Veterinary Medicine, Cairo University, Egypt. The animals were housed in metal wire mesh cages (3–4 rats per cage) and were left for 2 weeks before beginning the experiment for acclimatization. The housing conditions including temperature 25 ± 2 °C, relative humidity 50–60% and 12 h photoperiods were set. The rats were supplied with a pelleted diet and water *ad libitum*.

### Chemicals

Diethylnitrosamine (DENA) was purchased from Sigma Chemical Co. (St. Louis, MO). Phenobarbitone was kindly supplied by the Egyptian International Pharmaceutical Industry Co. (EIPICO). Cisplatin was purchased from El Azaby pharmacy under the commercial name of cisplatin 10 mg.

### Camel milk

Camel milk was purchased from a camel ranch in Ras Sedr, south of Sinai. Camel milk was analyzed using Milk Lactoscan in Department of Nutrition, Faculty of Veterinary Medicine, Cairo University.

### Experimental design

Fifty-six male rats were divided into eight groups (seven rats each).

*Group I* served as a negative control group.

*Group II* was treated with camel milk 5 mL by oral intubation daily after 28 weeks of experimental commence.

*Group III* was intraperitoneally injected with cisplatin (5 mg/kg) for two times with 3 weeks interval after 28 weeks of experimental commence.

*Group IV* was treated with camel milk 5 mL by oral intubation daily and was intraperitoneally injected with cisplatin (5 mg/kg) for two times with 3 weeks interval after 28 weeks of experimental commence.

*Group V* was injected intraperitoneally with a single dose (200 mg/kg body weight) of diethylnitrosamine (DENA) dissolved in saline to initiate hepatocarcinogenesis. After one week, phenobarbitone was added to drinking water at a concentration of 0.05% (500 ppm) for 27 weeks.

*Group VI* was injected intraperitoneally by a single dose of DENA (200 mg/kg BW) and after one week, phenobarbitone was added to drinking water at a concentration of 0.05% (500 ppm) for 27 weeks. Treatment with camel milk (5 mL daily) began at the 28th week.

*Group VII* was injected intraperitoneally by a single dose of DENA (200 mg/kg BW) and after one week, phenobarbitone was added to drinking water at a concentration of 0.05% (500 ppm) for 27 weeks. Rats were then intraperitoneally injected with cisplatin (5 mg/kg) for two times with 3 weeks interval after 28 weeks.

*Group VIII* was injected intraperitoneally by a single dose of DENA (200 mg/kg BW) and after one week, phenobarbitone was added to drinking water at a concentration of 0.05% (500 ppm) for 27 weeks. Rats were then treated with camel milk (5 mL daily) and were intraperitoneally injected with cisplatin (5 mg/kg) for two times with 3 weeks interval after 28 weeks of experimental commence.

Three rats from each group were then euthanized using an overdose of chloroform after 34 weeks of DENA injection (6 weeks of camel milk treatment). The rest of the rats were euthanized after 38 weeks (9 weeks of camel milk treatment).

### Biochemical analysis

Blood was collected using capillary tubes from the medial canthus of the eye under anaesthesia using chloroform. The blood was then centrifuged at 3000 rpm for 10 min for separation of serum. Serum AST, ALT, albumin, total protein, urea and creatinine were analyzed spectrophotometrically using commercially available kits (Spectrum, Egypt).

### Estimation of superoxide dismutase and lipid peroxidation

The activity of superoxide dismutase (SOD) and concentration of malonaldehyde (MDA) were estimated in the liver homogenate made from frozen liver samples using Biodiagnostic Kit (Egypt) according to Marklund and Marklund (1974) and Ruiz-Larrea et al. (1994), respectively.

### Histopathology

Liver and kidney samples were fixed for 48 h in 10% neutral-buffered formalin and processed by paraffin embedding technique. Sections of 3–5 μm thick were prepared and stained with H&E stain for microscopic examination (Suvarna et al. 2012). Liver specimens of each rat were obtained from three different lobes and lesion score for altered hepatocellular foci was performed. Histopathological diagnosis of liver cell foci and neoplasms was performed according to the histological criteria of the Institute of Laboratory Animal Resources (Stewart et al. 1980). The mean area of foci was measured in group V, VI, VII and VIII using image analyzer Leica Quin 500 (Pathology department, National Research Center, Giza, Egypt). The mean area of foci was measured in 10 microscopic fields/3 tissue samples/group and statistical analysis of the data was carried out.

### Immunohistochemical staining of placental glutathione S-transferase (P-GST)

Paraffin-embedded liver tissue sections were immunohistochemically stained using anti-P-GST polyclonal antibody prepared in rabbit (MBL Co. ltd, Nagoya, Japan) and the avidin–biotin-peroxidase complex method according to kit manufacturer protocol (Dako, LSAB + system-HRP, North America, Inc., MI, USA). The mean area of P-GST positive foci were measured using image analyzer Leica Quin 500, Pathology department, NRC. The mean areas of enzyme altered foci per microscopic field (5×) were measured in 10 microscopic fields/3 tissue samples/group and the statistical analysis of the data was performed.

### Statistical analysis

Statistical analysis was carried out using statistical package SPSS, version 8.0 (SPSS Inc., Chicago, IL). Statistical analysis of data was carried out using one-way analysis of variance (ANOVA) followed by LSD and Duncan test. Results were expressed as a mean ± standard error (mean ± SE). *p* values less than 0.05 were considered significant.

## Results

### Biochemical analysis

At the 34th week, albumin significantly decreased in group VII injected with DENA and treated with cisplatin compared to other groups and also was decreased in group III injected with cisplatin but showed no significance. The ALT serum activity showed no significant alteration between groups. On the other hand, the AST activity was increased in group VIII injected with DENA and treated with camel milk and cisplatin compared to other groups except group VI injected with DENA and treated with CM. Concerning the urea, it was elevated in group VII injected with DENA and treated with cisplatin but recorded no significance with control (Table 1). Similarly, there was no significance in the creatinine concentration although there was a mild elevation in group VII and group VIII. On the other hand, the creatinine concentration significantly decreased in group II treated with CM and IV treated with CM and cisplatin compared to control. At the 38th week, the total protein markedly increased in group V injected with DENA only and the urea concentration was elevated in group VIII injected with DENA and treated with cisplatin and camel milk (Table 2). The ALT serum activity was almost comparable between groups however group VI showed a slightly significant increase in activity compared to group I and group V.

**Table 1.** Results of biochemical analysis performed on rat serum subjected to different treatments at 34th weeks post injection of DENA.

GP	Total protein (g/dL)	Albumin (g/dL)	ALT (U/L)	AST (U/L)	Urea (mg/dL)	Creatinine (mg/dL)
I	9.33 ± 2.25^a,b^	4.33 ± 0.24^b^	23.66 ± 1.33	23.33 ± 4.33^a^	37.79 ± 4.96^b,c^	0.90 ± 0.09^b,c^
II	11.56 ± 1.96^b^	4.4 ± 0.10^b^	23.66 ± 1.33	37.66 ± 1.66^a^	27.46 ± 2.17^a,b^	0.65 ± 0.03^a^
III	6.43 ± 1.35^a^	4.06 ± 3.33^b^	19.66 ± 2.6	23.66 ± 6.22^a^	40.13 ± 3.2^b,c^	0.8 ± 0.05^a,b,c^
IV	12.48 ± 1.24^b^	4.30 ± 1^b^	29 ± 0	38 ± 7^a^	37.39 ± 6.3^b,c^	0.69 ± 0.08^a^
V	10.53 ± 1.55^a,b^	4.33 ± 3.33^b^	22.33 ± 1.33	30.33 ± 1.27^a^	24.5 ± 1.27^a^	0.77 ± 0.01^a,b^
VI	7.8 ± 0.40^a,b^	4.36 ± 0.18^b^	21 ± 0	48 ± 9.6^a,b^	37.03 ± 3^b,c^	0.81 ± 0.04^a,b,c^
VII	12.26 ± 0.17^b^	3.13 ± 0.23^a^	26.33 ± 1.33	31.33 ± 2.6^a^	47.13 ± 3.94 ^c^	0.92 ± 0.02^b,c^
VIII	9.93 ± 0.88^a,b^	4.3 ± 0.15^b^	22.33 ± 3.5	65 ± 13.85^b^	35.03 ± 2.5^a,b,c^	0.98 ± 0.07^c^

All data presented as mean value (*n* = 3) ± standard error. Values bearing different superscripts (a,b,c) are significant at *p* < 0.05. Group I is a control negative group. Group II was treated with camel milk. Group III treated with cisplatin. Group IV treated with camel milk and cisplatin. Group V injected with DENA. Group VI injected with DENA and treated with camel milk. Group VII injected with DENA and treated with cisplatin. Group VIII injected with DENA and treated with cisplatin and camel milk.

**Table 2.** Results of biochemical analysis performed on rat sera subjected to different treatments at 38th weeks post injection of DENA.

GP	Total protein (g/dL)	Albumin (g/dL)	ALT (U/L)	AST (U/L)	Urea (mg/dl)	Creatinine (mg/dl)
I	9.29 ± 1.08^a^	3.1 ± 0.24	14.5 ± 2.8^a^	39 ± 7.9^b^	32.4 ± 1.12^a^	0.99 ± 0.14
II	10.11 ± 0.67^a,b^	3.4 ± 0.25	26.25 ± 5.2^b,c^	19.5 ± 5.8^a^	32.5 ± 2.4^a^	1.08 ± 9.2
III	12.8 ± 1.37^a,b,c^	2.7 ± 0.35	27.25 ± 2.25^b,c^	17.75 ± 2.1^a^	49.97 ± 9.2^b,c^	0.92 ± 8.8
IV	9 ± 2.09^a^	3.2 ± 1.0	30.66 ± 1.66^b,c^	16 ± 0^a^	35.43 ± 2.3^a,b^	0.92 ± 5.29
V	15.83 ± 5^c^	2.5 ± 5.7	21 ± 2.3^a,b^	24 ± 6.65^a,b^	47 ± 7.1^a,b,c^	1.05 ± 0.2
VI	10.47 ± 1.79^a,b^	3.2 ± 0.33	32.7 ± 1.25^c^	34.5 ± 8.83^a,b^	41.1 ± 2^a,b,c^	0.9 ± 4.65
VII	14.68 ± 3.08^b,c^	2.9 ± 0.24	24.25 ± 3.6^b,c^	18 ± 3.13^a^	46.45 ± 5.6^a,b,c^	0.94 ± 3.4
VIII	13.5 ± 1.79^a,b,c^	3.4 ± 0.21	25 ± 1.63^b,c^	26.5 ± 4.9^a,b^	54 ± 4.9^c^	0.96 ± 4.12

All data presented as mean value (*n* = 3) ± standard error. Values bearing different superscripts (a,b,c) are significant at *p* < 0.05. Group I is a control negative group. Group II was treated with camel milk. Group III treated with cisplatin. Group IV treated with camel milk and cisplatin. Group V injected with DENA. Group VI injected with DENA and treated with camel milk. Group VII injected with DENA and treated with cisplatin. Group VIII injected with DENA and treated with cisplatin and camel milk.

### Superoxide dismutase activity and lipid peroxidation

Both the MDA concentration and the SOD activity expressed a significant difference between groups at a 34th week but not at 38th week. The MDA concentration was highly elevated in group V injected with DENA only compared to group I the control negative group. Due to an error that occurred during measuring the MDA concentration in liver tissue of group IV, its result was excluded and was not tabulated. The SOD activity, on the other hand, was severely decreased in group VII injected with DENA and treated with cisplatin compared to all groups and moderately decreased in group V compared to group II treated with CM. The highest SOD activity was recorded in group II treated with CM followed by group IV treated with CM and cisplatin. Furthermore, SOD activity in groups III, VI, VIII was comparable to the group I (control). At the 38th week, the SOD activity was decreased in all groups compared to their counterparts at 34th week. Although no significance was recorded in SOD activity at 38th week, the highest activity was recorded in group VI injected with DENA and treated with CM (Table 3).

**Table 3.** Results of MDA concentration and SOD activity at the 34th and 38th week post injection of DENA.

	MDA (nmol/g tissue)		SOD (U/g tissue)	
GP	34 w	38 w	34 w	38 w
I	24.89 ± 7.25^a^	25.28 ± 3.49	531.07 ± 3.02^b,c^	409.03 ± 60.44
II	41.42 ± 21.22^a,b^	25.59 ± 3.85	580.30 ± 8.46^c^	317.88 ± 36.05
III	23.94 ± 0.12^a^	43.45 ± 17.18	530.02 ± 5.54^b,c^	389.79 ± 141.53
IV	—————-	36.98 ± 7.04	541.20 ± 12.70^b,c^	388.81 ± 146.73
V	68.95 ± 3.0^b^	71.06 ± 36.66	424.58 ± 88.81^b^	418.42 ± 115.92
VI	43.06 ± 12.72^a,b^	45.52 ± 20.85	536.31 ± 19.46b^b,c^	463.29 ± 66.16
VII	17.11 ± 4.1^a^	49.21 ± 6.61	311.45 ± 41.71^a^	358.77 ± 101.09
VIII	36.5 ± 13.35^a,b^	72.16 ± 25.35	540.5 ± 29.94^b,c^	207.16 ± 35.69

All data presented as mean value (*n* = 3) ± standard error. Values bearing different superscripts (a,b,c) are significant at *p* < 0.05.Group I is a control negative group. Group II was treated with camel milk. Group III treated with cisplatin. Group IV treated with camel milk and cisplatin. Group V injected with DENA. Group VI injected with DENA and treated with camel milk. Group VII injected with DENA and treated with cisplatin. Group VIII injected with DENA and treated with cisplatin and camel milk.

### Histopathology

**Figure 1.**
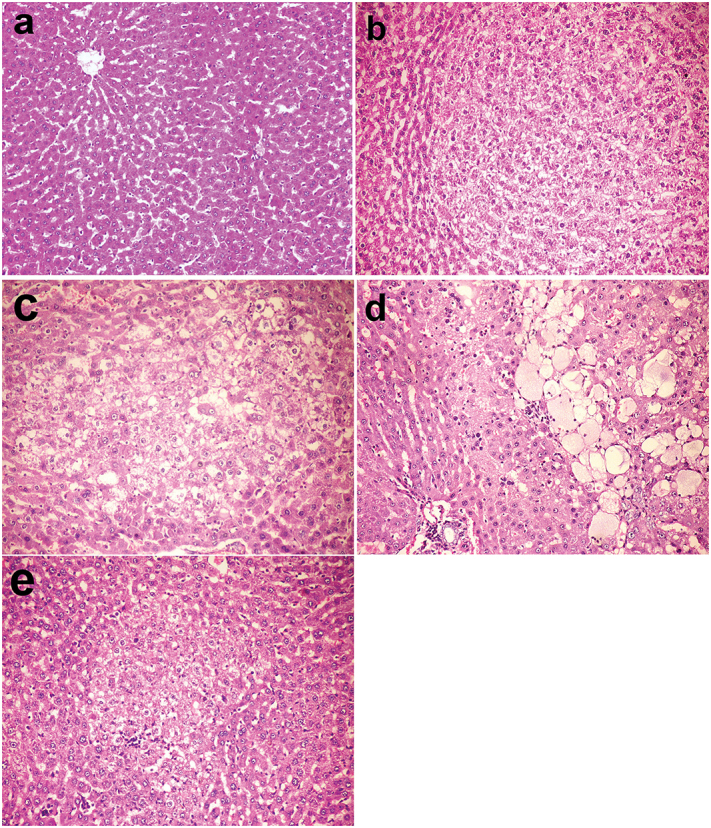
Livers, rats (38th week). (a) Normal hepatic structure in group I (control group). (b) Hepatocellular adenoma in group V injected with DENA. (c) Altered hepatocellular focus with decreased cellular density in group VI injected with DENA and treated with camel milk. (d) Hepatocellular adenoma with spongiosis hepatis in group VII injected with DENA and treated with cisplatin. (e) Mononuclear inflammatory cells invading the periphery of altered hepatocellular focus and single cell necrosis in group VI injected with DENA and treated with camel milk and cisplatin. Haematoxylin and eosin stain 200×.

The liver of rats in group I and II revealed almost no lesions (Figure 1(a)) whereas in group III and IV treated with cisplatin showed mild histopathological alteration in which eosinophilic cell infiltration was observed in the portal area. Nevertheless, the liver lesions were more severe in group VII injected with DENA and treated with cisplatin which was exemplified with the presence of portal haemorrhage.

**Figure 2.**
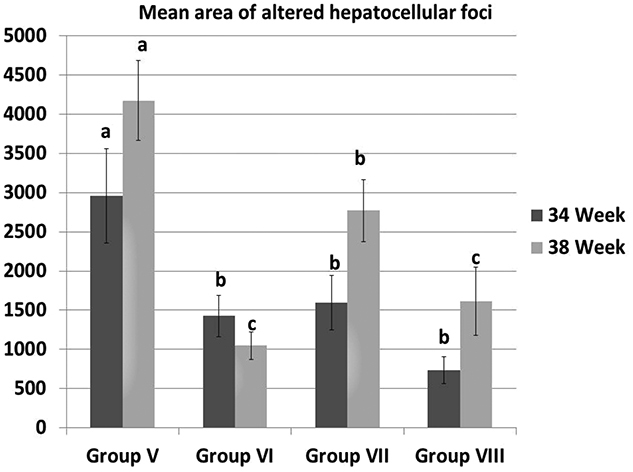
The mean area of altered hepatocellular foci in different groups at 34th and 38th week. All data were presented as mean value (*n* = 10) ± standard error. Values bearing different superscripts (a, b, c) are significant at *p* < 0.05.

The lesions observed in the liver of rats injected with DENA (group V) were degenerative changes in hepatocytes, few mononuclear inflammatory cells infiltration and biliary hyperplasia in the portal area, spongiosis hepatis and peliosis hepatis in addition to the presence of altered hepatocellular foci which were either comprised of clear cells, eosinophilic cells or basophilic cells. Hepatocellular adenoma and hepatocellular carcinoma also developed in this group (Figure 1(b)). All these previous lesions decreased in groups VI and VIII treated with camel milk (Figure 1(c–e)). The mean area of altered hepatocellular foci significantly decreased in groups VI, VII and VIII compared to group V at 34th week. Similarly, at 38th week, all treated groups showed a significant decrease in the mean area of hepatocellular altered foci compared to group V. However, group VI and group VIII recorded significant decrease not only compared to group V but also to group VII (Figure 2). Moreover, the liver of rats in group VIII developed no hepatocellular adenoma or carcinoma.

**Figure 3.**
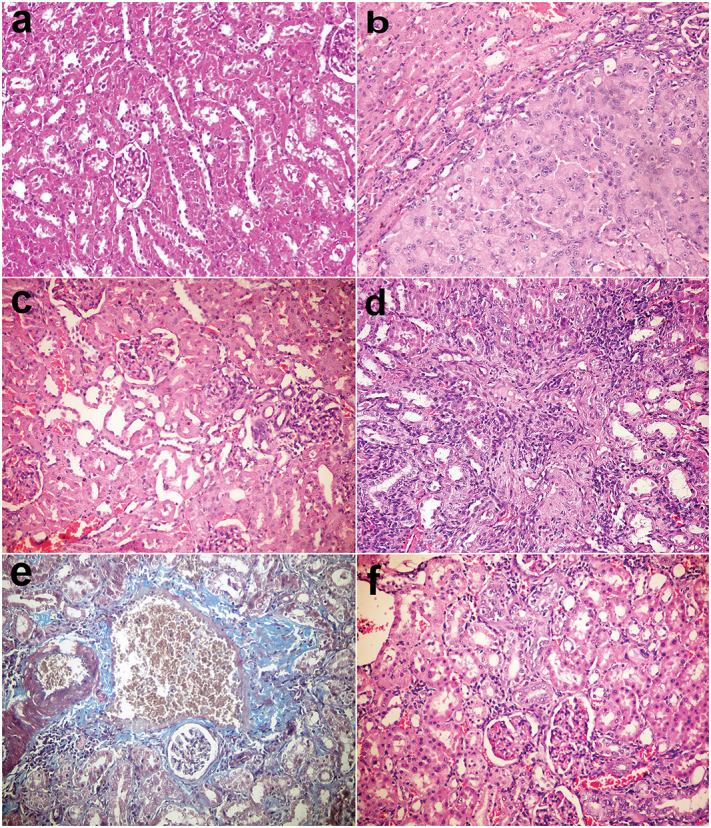
Kidneys, rats (38th week). (a) Normal renal tissue in group I control group. (b) Tubular cell adenoma of solid pattern with compression of adjacent parenchyma in group V injected with DENA. (c) Minor histopathological alteration in group VI injected with DENA and treated with camel milk. (d) Mononuclear inflammatory cells infiltration in the interstitial tissue with thickening of glomerular and tubular basement membrane and fibroplasia in group VII injected with DENA and treated with cisplatin. (e) Bluish-stained periglomerular and interstitial fibroplasia (Massons’ trichrome stain). (f) Few mononuclear inflammatory cells infiltration with regenerated renal tubules in group VI injected with DENA and treated with camel milk and cisplatin. Haematoxylin and eosin stain 200×.

In the kidneys, lesions were not observed in group I and II (Figure 3(a)). Lesions were demonstrated in group V injected with DENA in which tubular cell adenoma of solid pattern with compression of adjacent parenchyma was observed (Figure 3(b)). In the group injected with DENA and treated with camel milk, there was mild histopathological alteration (Figure 3(c)). Moreover, all groups injected with cisplatin revealed hypercellularity of capillary tuft with thickening of glomerular basement membrane, congestion of intertubular blood vessels, vacuolation and even necrosis of renal tubular epithelium in the renal medulla. There was also karyomegaly of the nuclei of tubular lining epithelium, interstitial nephritis, periglomerular and intertubular fibroplasia (Figure 3(d)) which gave positive with Masson’s Trichrome (Figure 3(e)) and cystic dilatation. However, the severity of these lesions was slightly alleviated in group IV and VIII treated with cisplatin and camel milk at a 34th week, but at 38th week the lesions were similar to other groups treated with cisplatin and showed severe nephropathy (Figure 3(f)).

### Immunohistochemical staining of P-glutathione-S-transferase

**Figure 4.**
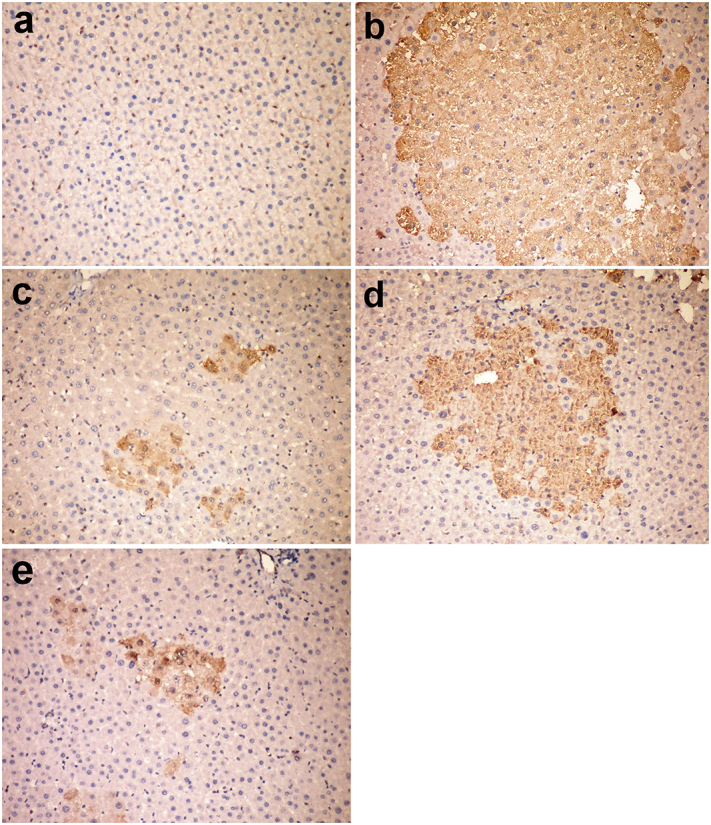
Liver, rat (38th week). **(a)** Negative staining for P-GST in group I (control group). **(b)** Large placental glutathione-*S*-transferase (P-GST) positive focus in group V injected with DENA. **(c)** Small-sized GST-P positive foci in group VI injected with DENA and treated with camel milk. **(d)** The medium-sized focus in group VII injected with DENA and treated with cisplatin**. (e)** Individual GST-P positive cells in group VI injected with DENA and treated with camel milk and cisplatin. Immunoperoxidase for GST-P 200 × (avidin–biotin–peroxidase complex method, Mayer’s Haematoxylin counterstain).

**Figure 5.**
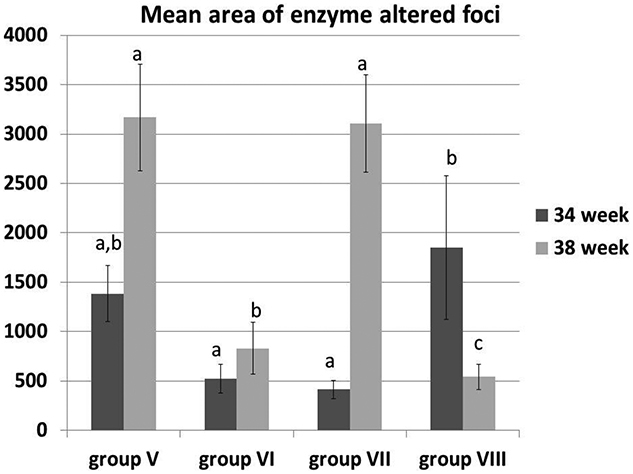
The mean area of enzyme-altered foci in the liver of different groups at 34th and 38th week. All data presented as mean value (*n* = 10) ± Standard error. Values bearing different superscripts (a, b, c) are significant at *p* < 0.05.

Large strongly positive enzyme altered foci were detected at 34th and 38th week in group V injected with DENA compared to the negatively stained control groups (Figure 4(a,b)). Group VII revealed the presence of large-sized enzyme altered foci which were almost comparable to group V (Figure 4(d)). On the other hand group, VI and group VIII showed small- and moderate-sized positively stained enzyme altered foci (Figure 4(c,e)). This variation was clearer at 38th-week post-injection of DENA than at 34th week. At 34 week, the mean area of enzyme-altered foci significantly decreased in group VI and VII compared to group VIII. However, at 38 weeks, group VI and VIII showed a significant decrease in the mean area of enzyme-altered foci compared to group V and VII (Figure 5).

## Discussion

Diethylnitrosamine initiates hepatocarcinogenesis through the interaction with DNA leading to mutation (Chakraborty et al. 2007) and enhanced production of free radicals causing oxidative stress (Valko et al. 2006). Promotion using phenobarbital has been implicated in several models of multistage hepatocarcinogenesis as quantified by the increase in number and size of enzyme-altered foci (Ito et al. 2003).

Reduced serum albumin in the group injected with DENA and treated with cisplatin has also been reported before (Saad & Al-Rikabi 2002). This reduction was not detected in the group treated with camel milk and cisplatin.

Experimental studies indicated that the maximum increase in serum creatinine occurs following cisplatin administration (Francescato et al. 2001). It was suggested that the increase in BUN and creatinine during cisplatin toxicity is due to irreversible renal tubular damage (Dickey et al. 2008). In the present study, there was no significant elevation in creatinine which could be due to the presence of 3 weeks intervals between injections which provided a chance for the creatinine level to be normalized.

ALT activity in the serum is increased in the case of hepatocellular injury or death due to the release of ALT from damaged liver cells (Kim et al. 2008). HCC usually favours the production of abnormal proteins in early stage but at a late stage, due to rapid progression, it could cause a marked ALT elevation. In the current study, the ALT level was not altered markedly in group V and other groups and therefore it was not used in the evaluation of camel milk and cisplatin.

The status of lipid peroxidation, as well as altered levels of certain endogenous radical scavengers, is taken as direct evidence for oxidative stress (Khan 2006). Diethylnitrosamine was found to cause elevation of lipid peroxidation levels and decreased levels of oxidative stress enzymes such as superoxide dismutase (SOD) and catalase in the liver tissue shortly after its injection in rats (Pradeep et al. 2007). Therefore, the elevation of MDA concentration in the group injected with DENA indicates increased lipid peroxidation and occurrence of oxidative stress in these groups. Moreover, the decrease in SOD activity in group VII indicates also the presence of oxidative stress in this group which could be related to the injection of DENA and cisplatin. On the other hand, the SOD activity was high and the MDA concentration was low in groups injected with DENA and treated with camel milk compared to group V injected with DENA. Consequently, it could be assumed that camel milk counteracted the oxidative stress induced by DENA in these groups.

At the 38th week, there was a significant elevation of total protein in group V although the albumin was not elevated which could be due to the ability of HCC to uniquely synthesize various tumour-related proteins which act as biomarkers (Behne & Copur 2012).

The altered hepatocellular foci, which are designated as preneoplastic (Farber 1984) are anticipated to grow into grossly visible nodules (Tatematsu et al. 1983). The size of these foci in the groups treated with CM in the present study was decreased and was almost similar at 34th and 38th week especially in the group treated with CM and cisplatin. The ability of CM to significantly inhibit the induction of the cytochrome P4501A1 (*Cyp1a1*), a cancer-activating gene, and to induce the NAD(P)H:quinone oxidoreductase 1 (*NQO1*), cancer chemopreventive gene (Korashy et al. 2012a) in addition to the activation of both the extrinsic and intrinsic apoptotic pathways (Korashy et al. 2012b) could be considered the main contributing factors in the improvement seen in these groups added to the antitumour properties of cisplatin (González et al. 2001).

Liver injury induced by chemicals such as nitrosamines encompasses hepatic vacuolization, necrosis, fibrosis, bile duct or hepatocyte hyperplasia and neoplasia (Boorman et al. 1990). Peliosis hepatis which is usually associated with hepatocellular neoplasms was believed to be induced by certain chemicals particularly nitrosamines (Boorman et al. 1990). Although the underlying mechanism of its formation is unclear it was thought to be a result of endothelial damage (Gushiken 2000). Moreover, spongiosis hepatis was reported following exposure to hepatocarcinogen (Boorman et al. 1990). It was believed that spongiosis hepatis is derived from altered perisinusoidal (Ito) cells in rat liver (Stroebel et al. 1995).

Camel milk was able to decrease the degeneration of some hepatocytes in rats with ethanol-induced liver injury (Darwish et al. 2012) and exerted a protective effect against the cytotoxicity of cisplatin (Salwa & Lina 2010). In the current study, the hepatocyte vacuolation, peliosis hepatis, spongiosis hepatis were remarkably absent in groups treated with CM. These lesions, however, increased in livers of rats treated with cisplatin which in turn emphasize on the hepatoxic effect of cisplatin on liver (Arhoghro et al. 2012).

Placental glutathione-*S*-transferases P-GST has been reported as a sensitive marker for preneoplastic hepatic foci during chemical hepatocarcinogenesis in rats (Qin et al. 1998; Guo et al. 2000). P-GST expression in a single hepatocyte promotes cell proliferation as well as its own expression in the adjacent cells (van Gijssel et al. 1997). In the present study, the mean per cent area of enzyme altered foci decreased in groups treated with CM. The positive effect exhibited by CM was potentiated with cisplatin as evidenced by the reduced mean per cent area of enzyme-altered foci in group VIII.

In the current study, kidneys of few rats injected with DENA showed tubular hyperplasia and tubular cell adenoma. Although DENA is a potent hepatotoxin and liver is the main target organ, a single dose of DENA was found to have a carcinogenic effect on the renal tubular epithelium causing tubular hyperplasia, tubular cell adenoma and tubular cell carcinoma (Athar & Iqbal 1998). Moreover, cisplatin is known to cause severe nephrotoxicity which is the most important dose-limiting factor (Ramya et al. 2013). Consequently, these lesions observed in groups treated with cisplatin were attributed to the direct effect of cisplatin. Different mechanisms have been implicated in cisplatin nephrotoxicity including the expression of glutamyl transpeptidase (Hanigan & Devarajan 2003), the initiation of apoptotic pathways (Cummings & Schnellmann 2002) and generation of reactive oxygen metabolites (Ueda et al. 2000).

## Conclusions

Camel milk possessed a good therapeutic potential which was potentiated with cisplatin. This positive therapeutic effect was believed to be due to the improvement observed in the superoxide dismutase activity and reduction of the lipid peroxidation in addition to decreased mean area of altered hepatocellular foci and mean area per cent of P-GST positive foci. Moreover, camel milk was able to relatively alleviate the renal side effect of cisplatin.
